# Competency of triage nurse in the emergency department: A scoping review protocol

**DOI:** 10.1371/journal.pone.0331982

**Published:** 2025-09-09

**Authors:** Miao Yu, Li Ma, Qingbian Ma

**Affiliations:** Emergency Department, Peking University Third Hospital, Beijing, China; King Saud University Medical City, SAUDI ARABIA

## Abstract

**Introduction:**

Triage is an essential strategy to mitigate crowding and guarantee patients’ safety in emergency departments. To improve the quality of triage in emergency departments, Nurses should be equipped with the necessary competencies. Therefore, this review aims to synthesize available evidence on the competency elements required for triage nurses in emergency departments and to identify factors that influence their competency development.

**Methods and analysis:**

This scoping review will be implemented following the five steps outlined by Arksey and O’Malley. We will use the PCC (population, concept, context) frameworks-Triage nurse (Population), Nursing competency (Concept), and EDs (Context)- to determine the research questions, and formulate the search terms. We will search six electronic databases including PubMed, Embase, CINAHL Plus, Web of Science, and two Chinese databases (China National Knowledge Infrastructure and Wangfang Data). Internet resources including WorldCat, and Google Books will be also searched to ensure comprehensive coverage. Studies will be selected by two independent authors based on defined eligibly criteria, and completed in August 2025. This will be followed by data extraction, and summarizing in October 2025. Then, evidence will be synthesized using descriptive statistics and thematic analysis. Five-domain Consolidated Framework for Implementation Research will be used to guide our thematic analysis of barriers and facilitators to development of competency. The results will be presented in December 2025. Findings from this scoping review will be beneficial to develop the training programs to facilitate the successful transition of nurses into effective triage nurse roles in the future.

**Registration:**

The scoping review was registered in Open Science (https://osf.io/6fcr4).

## Introduction

Overcrowding in emergency departments (EDs) has emerged as a global public health issue due to the excessive demand for medical services and the relative scarcity of resources [1]. It is estimated that 144.82 million EDs visits occurred in the United States, with a cumulative growth of 12.29% from 2010 to 2016 [2]. To alleviate overcrowding and ensure patient safety, a triage procedure is the first to be implemented in EDs before a formal doctor consultation. Triage refers to a decision-making process that allocates patients to the most appropriate assessment or treatment area within an appropriate waiting time based on the severity of condition [3]. The evidence indicated that rapid and accurate triage could minimize patients’ waiting time, and reduce mortality rate [4,5].

Nursing competency can be defined as the combination and application of nurses’ knowledge, professional skills, judgments, values, and attitudes, based on scientific knowledge and expectations of nursing practice in specific contexts [6]. With the development of triage, nurses play an increasingly important role, whether in a nurse-led triage or in team triage [7,8]. They are expected to possess competencies-such as advanced triage, resource and workflow management, crowding mitigation strategies, teamwork, and efficient clinical decision-making-specifically to address the critical imbalance caused by patient demand exceeding available staff, space, and resources within the ED, thereby reducing delays and improving care quality. However, evidence showed that providing high-quality of triage remains a challenge for nurses in EDs [9–11]. In an evidence-based literature review including 14 studies, it is reported that the accuracy of triage varies considerably, ranging from 59.3% to 82% [12]. It is likely that the majority of triage nurses had not received formal training to develop those necessary competencies [13,14]. Thus, the clarification of triage competencies is essential to identify, guide and train triage nurses to meet the requirement in EDs [15,16].

Originally, previous publications have primarily focused on clinical skills of triage such as decision-making, and communication skills [17–19]. With the development of triage sub-specialty, triage nurses should not only master triage skills, but also adhere to ethical principles, have legal awareness, maintain emotional stability and pay attention to their own professional development [20–22]. However, no comprehensive review has systematically mapped the full spectrum of competencies required for effective triage nursing. Meanwhile, the factors influencing the competencies of triage nurses have been discussed in different literatures but these factors have typically not been clearly synthesized into a holistic framework. Researchers can use this model to analyze factors multidimensionally, providing a framework for exploring the competency of triage promotion. Therefore, we aimed to firstly synthesize available evidence about the elements of competencies relevant to triage nurses in EDs, and identify factors that can affect the competency of triage promotion among nurse

## Materials and methods

### Protocol design

This scoping review will be conducted using the framework outlined by Arksey and O’Malley [23]. The framework included the following five stages: Step 1, determining the research questions; Step 2, identifying relevant studies; Step 3, study selection; Step 4, charting the data; and Step 5, collating, summarizing, and reporting the results. The publication was reported adhering to the Checklist Preferred Reporting Items for Systematic Reviews and Meta-Analyses extension for Scoping Reviews (PRISMA-ScR) [24]. The PRISMA-ScR checklist is presented in S1 Table.

**Fig 1:**
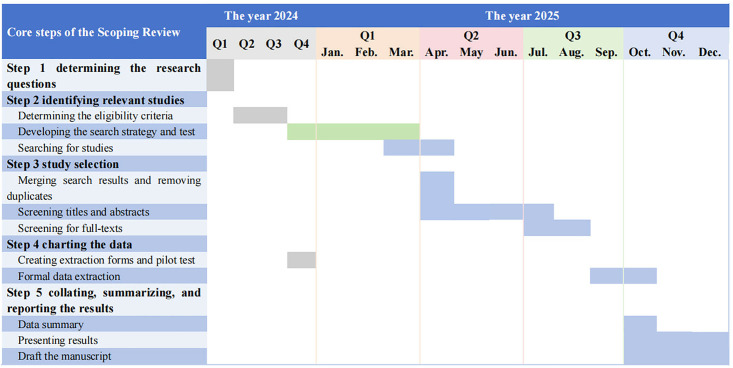
The timeline of the sudy. Note: Grey = Done. Green = In Progress. Blue = To Do. Q1, first quarter of the year; Q2, Second quarter of the year; Q3, third quarter of the year; Q4, fourth quarter of the year.

The scoping review was registered in Open Science Framework (https://osf.io/6fcr4). The formal record screening of all planned databases will be completed in August 2025. Data extraction will be completed in October 2025. The results are expected to be presented in December 2025. The detailed timeline of the study is shown in Fig 1.

**Step 1: determining the research questions**

The research questions were formulated by the research team, which included three nurses and two doctors, who had worked in the EDs for more than 10 years and had professional knowledge in triage. We used the PCC (population, concept, context) frameworks as follows: Triage nurse (Population), Triage competency and its influencing factors (Concept), and EDs (Context).

(P)opulation – Triage nurse. Any types of licensed nurse who performed triage process in EDs will be studied, such as Nurse Practitioners (NPs), Advanced Nurse Practitioner (ANP), and Emergency Nurse Practitioner (ENP).

(C)oncept- Triage competency and its influencing factors. Triage competency refers to a complex integration of knowledge, professional skills, judgments, values and attitudes based on scientific knowledge and expectations of nursing practice in specific contexts [3,6], such as clinical assessment, decision-making, documentation, communication skills, legal and policy awareness, and cultural sensitivity and ethics. We will also focus on the factors that promote or inhibit triage competency development.

(C)ontext- EDs. In this setting, medical resources are relatively abundant, but not every patient can get services immediately. Relatively scarce resources need to be reasonably allocated to unlimited medical needs [25,26]. EDs in public tertiary in urban will be included.

Consequently, the research questions for this review were:

(1) What are the frequently mentioned **competencies** of **triage nurse** in **EDs**?
(2) What are the **factors** influencing the **competencies** of **triage nurse** in **EDs**?

**Step 2: identifying relevant studies**

### Eligibility criteria

The detailed inclusion and exclusion criteria are shown in Table 1. To better illustrate the questions of interest, and facilitate the eligibility criteria, we used the PCC (population, concept, context) framework.

**Table 1:** Eligibility criteria.

Domain	Inclusion	Exclusion
Population	Licensed nurse who engaged in triage activities. They can perform triage independently or play a role in a triage team	Medical students
Content	Triage competency required for adults (aged ≥18 years)	Did not explain what specific aspects of triage competency
	Triage competency mentioned in standardized tools or validated local triage tools, including those used in specialized contexts (e.g., fast-track systems, mass casualty incidents, or disaster response)	Triage of special patient subgroups or specific condition (e.g., pregnancy, cancer)
Context	Emergency departments	Pre-hospital settings, outpatient departments, non-face-to-face triage, free-standing care or urgent care
Type of study	Policy documents, research articles, reviews, qualitative research, conference abstracts, dissertations, and books	editorials, commentaries, study protocols or did not have full-text availability
Language	English or Chinese	
Publication year	From January 1, 2015	

### Search strategy

With the assistant of a research librarian, six electronic databases including PubMed, Embase, CINAHL plus, Web of Science, and two Chinese databases (China National Knowledge Infrastructure and Wangfang Data) will be searched from January 1, 2015 onwards to capture the latest 10 years’ key competencies within the contemporary EDs context. Internet resources including WorldCat, and Google Books will be also searched to 4. to ensure comprehensive coverage.

Search terms will be derived from the PCC (population, concept, context) elements, incorporating Medical Subject Headings (MeSH), relevant keywords, synonyms, and truncation, combined using Boolean logic. The three key terms were triage, emergency department, and competency. The strategy will be developed iteratively and piloted. The pilot search strategies developed for PubMed is shown in S2 Table. Reference lists of included studies will be screened to supplement database searches.

**Step 3: study selection**

All searched studies will be exported into EndNoteX9 (Clarivate Analytics). After elimination of duplicates, two independent authors (M. Yu, and L. Ma) identify potentially relevant evidence beginning with a review of the titles and abstracts adherence to the eligibility criteria to ensure appropriate exclusion of irrelevant, outdated or opinion-based literature (Table 1). Then full-text of remaining studies will be retrieved and further evaluated by two independent authors (M. Yu, and L. Ma). Researchers review the reference lists of the included studies and added relevant studies. The study selection process will be presented in line with the PRISMA for Scoping Reviews flow diagram [27]. Any disagreements in each selection step will be resolved by discussion with a third author (Q. Ma).

**Step 4: charting the data**

The data extraction form based on Microsoft Excel will be piloted with two authors on five studies (Table 2). Data extraction employed the five-domain Consolidated Framework for Implementation Research (CFIR) to systematically categorize determinants of implementation success. This structured approach guided our thematic analysis of barriers and facilitators to development of competency across: 1) Intervention characteristics, 2) Outer setting, 3) Inner setting, 4) Individual characteristics, and 5) Implementation processes. The framework’s application ensured consistent coding of qualitative findings and informed our development of practice recommendations. [28–30]. Minor adaptations were made to specifically to describe the content of triage competency including study characteristic, competency, and features of competency assessment tools (if application).

**Table 2:** Description of the collected data.

No.	Sections	Description
**Literature general information**		
1	Study ID	Article were coded with letter ‘A’ followed by three digits (e.g., A001)
2	Author	
3	Publication year	
4	Country	
5	Type of paper	A review paper, commentary, editorial, qualitative study, quantitative study (observational study, experimental study, ……) or other forms
**Study characteristics**		
6	Study design	
7	Population	Sample size/ Demographic information (gender, and age)
**Competency**		
8	Identify number	Each competency was coded with letter ‘C’ followed by three digits (e.g., C001)
9	Description	Name and the explanation of each competency identified in the research.
**Features of competency assessment tools (if application)**		
10	Assessment tool	Name of assessment tools.
11	Response options	The potential answers the author provided to the respondents, such as a Rating Scale (a Likert scale), a binary, open-ended field, or other format.
12	Interpretation	Explanation of the score.
13	Items/ domains	Number and description of items and domains in the assessment tool.
14	Psychometric properties	Validity and reliability
**Identified factors that influencetriage competency**		
15	Innovation	
16	Outer setting	
17	Inner setting	
18	Characteristics of individuals	
19	Implementation process	

The group discussion will be conducted to the finalize the standardized form framework before formal data extraction. The relevant data will be extracted by two independent authors. Any disagreement in the process of data extraction will be resolved by group discussion. The group will consist of five core research team members with expertise in triage. The lead investigator will organize offline meetings to discuss the items in the preliminary draft extraction form or any disagreement in the extraction process until a consensus is reached on all aspects.

**Step 5: collating, summarizing, and reporting the results**

After charting the data, in order to describe the content of triage competency, and count for the frequency of repetition, we will code the article with letter ‘A’ followed by three digits (e.g., A001), and each competency will be coded with letter ‘C’ followed by three digits (e.g., C001). Therefore, for example, the competency encoded as ‘C020 A012’ can be identified as: competency 20 belonging to article 12. Then we will count for frequency of repetition. Finally, three authors will classify the competences according to their common characteristics, and rename the groups. Each category will be coded with the letter “G” followed by three digits (e.g., G001).

In the collating process, any disagreement will be resolved by our study group discussion. Findings from this scoping review will be beneficial to develop the training programs to facilitate the successful transition of nurses into effective triage nurse roles in the future. Any changes to the protocol will be reported in the final scoping review.

## Discussion

Triage is an essential strategy for mitigating emergency department (ED) crowding and ensuring patient safety, requiring nurses to possess specific competencies [31–33]. This scoping review will address a critical gap by synthesizing reported triage competencies and identifying factors affecting their development with several strength. We will employ the PCC framework to structure research questions, ensuring conceptual rigor, and adopt the CFIR framework to systematically synthesize factors influencing competency development. Furthermore, our evidence base extends beyond periodicals and grey literature to include authoritative scholarly books, capturing theoretical depth alongside empirical evidence.

The results of our review will provide actionable insights for improving emergency care systems. At the policy level, these findings can inform guideline updates the competencies of triage nurse to further promote the professional growth. For clinical practice, we identify evidence-based pathways to enhance staff training programs, and overcome training program implementation barriers [34]. However, only articles in English and Chinese will be included, which might limit our capacity to identify all eligible studies. Given the scoping nature of this scoping review-focused on identifying the range of research rather than synthesizing high-quality evidence, we will not conduct formal quality appraisal. This may affect reality of interpretation of the findings. In the future, assessment of the quality of literature are required in systematic review.

## Supporting information

S1 Table Preferred Reporting Items for Systematic Reviews and Meta-Analyses extension for Scoping Reviews (PRISMA-ScR) Checklist. (DOCX)

S2 Table Pilot search strategy in PubMed. (DOCX)
