# Supplementary material for: Competency of triage nurse in the emergency department: A scoping review protocol
Source: PLoS One. 2025 Sep 9;20(9):e0331982. doi: 10.1371/journal.pone.0331982 (PMC12419642; doi:10.1371/journal.pone.0331982)
Supplement: S2 Table — (DOCX) [file pone.0331982.s002.docx]

**Supplemental Table S2 Pilot search strategy in PubMed**

| **Database** | **Search strategies** | **Results** |
| --- | --- | --- |
| PubMed | #1 "triage"[MeSH Terms] OR "triage"[Title/Abstract] OR "overtriage"[Title/Abstract] OR "undertriage"[Title/Abstract] OR "classification"[Title/Abstract] | 524,648 |
|  | #2 "emergency service, hospital"[MeSH Terms] OR "emergency medical services"[MeSH Terms] OR "emergency nursing"[MeSH Terms] OR "emergency medicine"[MeSH Terms] OR "hospitals"[MeSH Terms] OR "Emergenc*"[Title/Abstract] OR "hospital*"[Title/Abstract] OR "emergency nurs*"[Title/Abstract] | 2,427,325 |
|  | #3 "clinical competence" [MeSH Terms] OR "professional competence"[MeSH Terms] OR "competenc*"[Title/Abstract] OR "skill*" [Title/Abstract] | 478,925 |
|  | #4 #1 AND #2 AND #3 | 2,161 |
